# Prediction of Robotic Anastomosis Competency Evaluation (RACE) metrics during vesico-urethral anastomosis using electroencephalography, eye-tracking, and machine learning

**DOI:** 10.1038/s41598-024-65648-3

**Published:** 2024-06-25

**Authors:** Somayeh B. Shafiei, Saeed Shadpour, James L. Mohler, Parisa Rashidi, Mehdi Seilanian Toussi, Qian Liu, Ambreen Shafqat, Camille Gutierrez

**Affiliations:** 1 Intelligent Cancer Care Laboratory, Department of Urology, Roswell Park Comprehensive Cancer Center, Elm and Carlton Streets, Buffalo, NY 14263 USA; 2 Department of Animal Biosciences, University of Guelph, Guelph, ON N1G 2W1 Canada; 3 Department of Urology, Roswell Park Comprehensive Cancer Center, Buffalo, NY 14263 USA; 4 Department of Biomedical Engineering, University of Florida, Gainesville, FL 32611 USA; 5 Department of Biostatistics and Bioinformatics, Roswell Park Comprehensive Cancer Center, Buffalo, NY USA; 6 Obstetrics and Gynecology Residency Program, Sisters of Charity Health System, Buffalo, NY 14214 USA

**Keywords:** Radical prostatectomy, Vesico-urethral anastomosis, Surgical performance, Quantitative assessment, Urological cancer, Biomedical engineering

## Abstract

Residents learn the vesico-urethral anastomosis (VUA), a key step in robot-assisted radical prostatectomy (RARP), early in their training. VUA assessment and training significantly impact patient outcomes and have high educational value. This study aimed to develop objective prediction models for the Robotic Anastomosis Competency Evaluation (RACE) metrics using electroencephalogram (EEG) and eye-tracking data. Data were recorded from 23 participants performing robot-assisted VUA (henceforth ‘anastomosis’) on plastic models and animal tissue using the da Vinci surgical robot. EEG and eye-tracking features were extracted, and participants’ anastomosis subtask performance was assessed by three raters using the RACE tool and operative videos. Random forest regression (RFR) and gradient boosting regression (GBR) models were developed to predict RACE scores using extracted features, while linear mixed models (LMM) identified associations between features and RACE scores. Overall performance scores significantly differed among inexperienced, competent, and experienced skill levels (*P* value < 0.0001). For plastic anastomoses, R^2^ values for predicting unseen test scores were: needle positioning (0.79), needle entry (0.74), needle driving and tissue trauma (0.80), suture placement (0.75), and tissue approximation (0.70). For tissue anastomoses, the values were 0.62, 0.76, 0.65, 0.68, and 0.62, respectively. The models could enhance RARP anastomosis training by offering objective performance feedback to trainees.

## Introduction

Urethral anastomosis, a key step in radical prostatectomy, is incorporated into early residency training due to its significant impact on patient outcomes^1^. Therefore, the assessment and training curriculum for this procedure hold substantial educational value. The posterior or posterolateral part of the anastomosis is challenging to construct due to its placement deep in the pelvis^2^. Incomplete apposition may result in urinary leakage, whereas extremely tight suturing may reduce blood flow and cause contracture. The incidence of postoperative anastomotic/bladder neck contracture (BNC) after radical prostatectomy was reported to be up to 2.2% in a study from 2002 to 2008 of 988 patients^3^. Patients with BNC suffer from a poor urinary stream or prolonged incontinence. As a result, it is crucial to avoid anastomotic leaks and hematoma during urethral anastomosis.

The majority of BNC are believed to result from poor anastomotic technique. Watertight closure and meticulous mucosal apposition may avoid urine leakage^4^. Prolonged urine leakage caused by an anastomotic gap may result in scarring and contracture at the bladder neck. Operative bleeding or traction on the anastomosis may cause tissue ischemia and increase the chance of BNC formation^5,6^. These factors suggest that surgeons should become competent in performing an anastomosis before performing the procedure on a patient.

The da Vinci Surgical System (Intuitive Surgical, Inc., Sunnyvale, CA) is being used in operating rooms (OR) worldwide to perform anastomosis. This system provides three-dimensional vision, seven degrees of freedom, and magnification. However, robotic surgical skills acquisition is costly and requires learning human–computer interaction^7^. A surgeon must be skilled in synchronized hand movements, three-dimensional spatial orientation, depth perception, and the use of magnification to perform robot-assisted surgery (RAS)^7^.

In RAS training programs, experienced surgeons typically subjectively assess trainees’ performance in performing anastomosis. An expert must remain present and attentive throughout the session. Their assessment is prone to bias since each experienced surgeon may give different weights to different aspects of performance. A performance evaluation that removes these inconsistencies is referred to as an “objective performance assessment”. There are benchmarked and quality-assured global training standards for the aviation industry^8^. The pilot must demonstrate proficiency in performance before a pilot is allowed to operate a plane. Surgical training has not yet used the same strict methodology^8^. An objective performance evaluation model is necessary to increase the effectiveness of training and give trainees objective feedback on how well they perform anastomosis.

Several studies have proposed objective surgical performance evaluation approaches using physiological data, such as eye movement^9,10^. Even fewer use EEG data to evaluate surgical performance and competence^11^. EEG is a neuroimaging technique that monitors brain activity directly and provides information about cognitive states that physiological and eye movement data cannot^12^. EEG has excellent temporal resolution, often measured in milliseconds, and can track changes in brain activity in real time. Physiological and eye movement data also provide real-time information, but they may not capture cognitive shifts as quickly or accurately. EEG has been used to evaluate performance in driving and aviation^13^. The use of EEG and eye-tracking for performance and skill levels evaluation in RAS anastomosis training is currently lacking.

Machine learning methods for objective performance evaluation have been frequently trained using information gathered from physiological data^14^. The suggested approaches showed promising results and ushered in a new era of objective surgical performance assessment. Small participant numbers and/or computationally costly models that cannot be integrated into surgical robot systems are some of the drawbacks of those approaches^14^. Furthermore, none of the suggested techniques were validated to work for RAS anastomosis.

The goal of this study was to develop machine learning models for predicting the performance of RAS anastomosis using EEG and eye-tracking features.

## Methods

This study was conducted in accordance with relevant guidelines and regulations approved by the Institutional Review Board (IRB; I-241913) of Roswell Park Comprehensive Cancer Center. The documentation of written consent was waived by the IRB and participants provided verbal agreement to participate.

### Data recording

**Figure 1:**
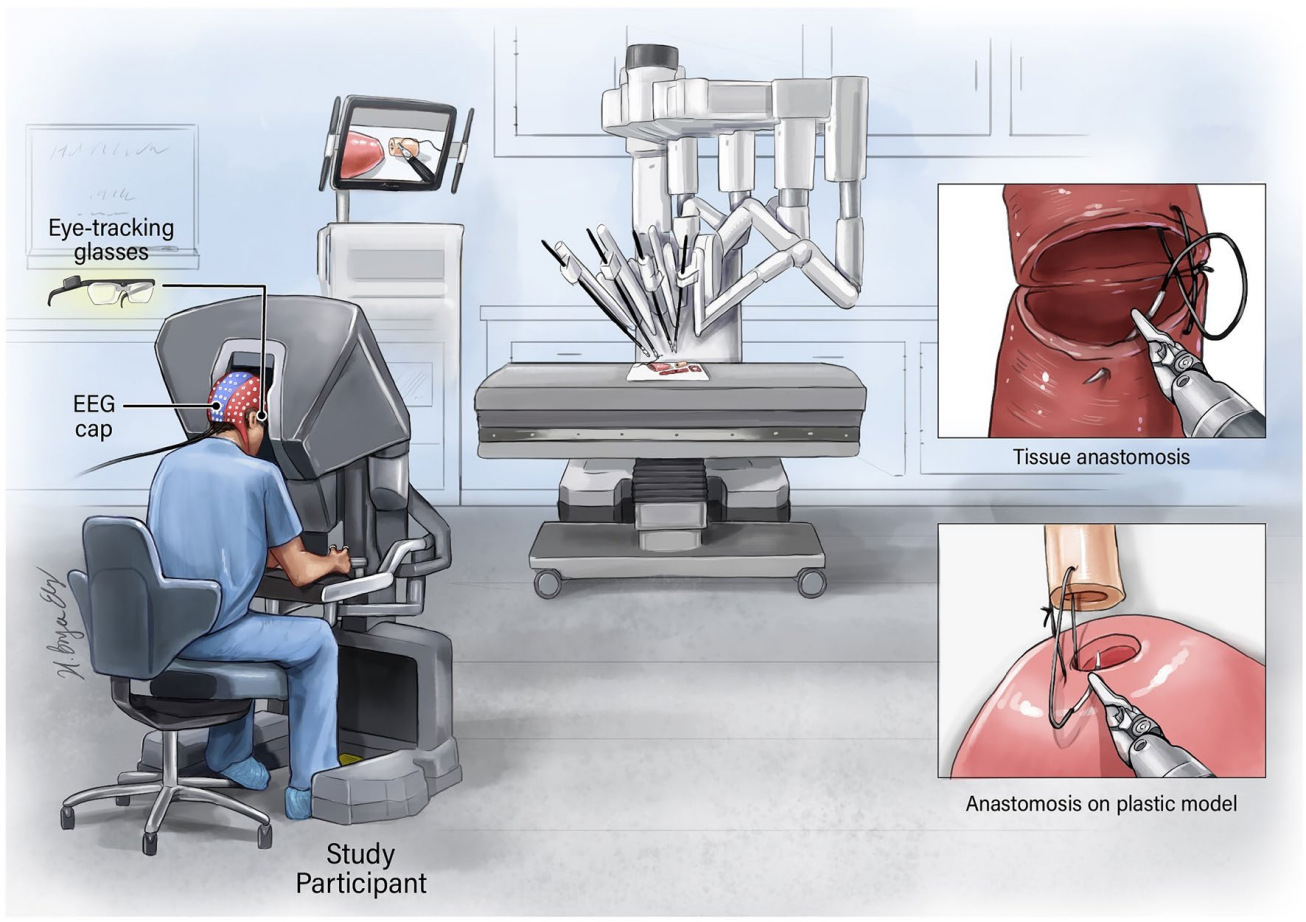
Representation of experimental setup. EEG and eye-tracking data were acquired while participants used the da Vinci robot to perform an anastomosis on plastic or animal tissue.

Eye-tracking data were recorded from participants using TobiiPro2 eyeglasses at a frequency of 50 Hz. EEG data were recorded from 124 locations on the scalp using an EEG headset (AntNeuro ©) at a frequency of 500 Hz, with Cz lead as the reference channel (Fig. 1). Videos were recorded during task conduction.

### Participants

Twenty-three right-handed participants (nine pre-medical students, two scientists, three residents, four fellows, and five surgeons) with varying hours of RAS experience performed two anastomoses on a plastic model and two tissue anastomoses on either porcine esophagus or intestine (Fig. 1). The ages of participants ranged from 22 to 67 (35 ± 12) years. Participants included 16 males and seven females.

### Vesico-urethral anastomosis

Participants performed anastomoses by suturing outside-to-inside on the bladder and inside-to-outside on the urethra (Fig. 1)^15^.

### Visual features

The eye-tracking data were processed using Tobii Pro Lab ©. A moving average filter with a window size of three points was used to decrease noise in the eye-tracking data. Fixation and saccadic time points were identified using a velocity-threshold filter with a threshold of 30° per second. The features derived from eye-tracking data included^10^: (F1) the average pupil diameter of nondominant eye; (F2) the average pupil diameter of dominant eye; (F3) Shannon entropy of nondominant eye’s pupil diameter; (F4) Shannon entropy of dominant eye’s pupil diameter; (F5) the rate of fixation; (F6) the rate of the saccade. The total number of visual features extracted is six, represented by F1–F6.

Eye-tracking features provide information about various aspects of visual perception and cognitive processing. Rate of fixation refers to the proportion of time spent fixating on a particular area of interest, which indicates the level of attention or interest in that area. Rate of the saccade measures the frequency of rapid eye movements between different areas of interest, which reflects the level of exploration and scanning of visual stimuli^16^. Average pupil diameter is a measure of the size of the pupil, which can reflect changes in cognitive and emotional processing^17^. Shannon entropy of pupil diameter is a measure of the variability of the pupil size over time, which can provide information about the level of arousal or cognitive load^18^. For each participant, the mean (µ) and standard deviation (σ) of each feature (X) within task attempts were calculated. By standardizing each feature value ((X − µ)/σ), the data were normalized for individual differences, similar to baseline correction^19,20^.

### EEG analysis

Due to poor signal quality, the electrodes F8, POz, AF4, AF8, F6, FC3, and the mastoid electrodes M1 and M2 were excluded from this study. Signal processing techniques previously described in our publications^20,21^, were applied to the EEG signals to remove noise^22–38^. Using the advanced source analysis (ASA) framework by ANT Neuro Inspiring Technology Inc., Netherlands, the remaining signals from 116 EEG channels were cleansed of artifacts through a process that integrated blind source separation with topographical Principal Component Analysis (PCA). The decontamination of EEG data involved five steps:

1. Re-referencing the EEG data to the ‘common average reference’ of all included channels.
2. Applying a 60 Hz notch filter to eliminate line noise artifacts.
3. Using a band-pass filter (0.2–250 Hz) with a 24 dB/octave steepness to filter the EEG data.
4. Visually inspecting specific EEG segments for artifacts from facial and muscular activities and cleaning these segments.
5. Employing a spatial Laplacian approach to emphasize sources at small spatial scales and mitigate the impact of volume conduction on coherence calculations.

The decontaminated EEG data were then analyzed to extract features related to strength, search information, temporal network flexibility, integration, and recruitment^20,21^.

### EEG feature extraction process

EEG channels were assigned to corresponding brain lobes (frontal, parietal, occipital, and temporal) based on the placement of recording electrodes, using the Brodmann Interactive Atlas^25,39^. The channels were then processed to extract various features using methods also employed in our previous research^20,21^.

- Search Information: Calculates the information (in bits) needed to navigate the most efficient path between network nodes. The Adjacency matrix described each EEG channel’s connectivity, and the Brain Connectivity Toolbox was used for extraction^26–28,36,40^. The adjacency matrix, representing the strength of connections between EEG channels, was calculated using coherence analysis to assess the synchrony between different brain regions.
- Temporal Network Flexibility: Assesses the frequency of nodes changing community groups over time^32^. Temporal flexibility was calculated using the flexibility function of the Network Community Toolbox^36^. Temporal flexibility was calculated for each lobe by averaging channel scores, indicating the stability or variability of community assignments.
- Strength Feature: Measures the degree of interconnectivity within each lobe, reflecting overall activity and connectivity^31^.
- Integration and Recruitment: Represent how nodes associate within and across lobes. These features were extracted using the Network Community Toolbox^36–38^.

The average of features was calculated for four different lobes of the brain: frontal, parietal, occipital, and temporal, resulting in 20 EEG features, labeled F7–F26. The same five types of measurements were applied across each lobe:

- Temporal network flexibility (F7, F12, F17, F22)
- Integration between channels in the cortex and other cortices (F8, F13, F18, F23)
- Recruitment of channels (F9, F14, F19, F24)
- Search information for channels (F10, F15, F20, F25)
- Strength of channels (F11, F16, F21, F26)

Each feature type was calculated for the parietal (F7–F11), frontal (F12–F16), occipital (F17–F21), and temporal (F22–F26) cortices, respectively.

EEG features provide information about how the brain processes information during an operation. Search information reveals how efficiently different brain regions communicate with each other^26–28^. Temporal network flexibility helps understand how the brain changes over time as it responds to various demands^32,36^, while integration describes how different brain regions collaborate over time^37^. When cognitive or behavioral activities are performed, recruitment refers to the activation of specific brain regions that form interrelated networks. These recruitment patterns offer information about the fundamental neural mechanisms behind diverse cognitive functions and provide an understanding of how the brain processes information and produces behavior^41,42^.

### Actual RACE metrics’ scores

Three raters, two RAS surgeons (J.L.M. and C.G.), and a data coordinator who was trained to perform and score these tasks (M.S.T.) used the RACE metrics to assess the surgical performance of the participants. RACE is a tool for assessing how well a RAS surgeon performs anastomosis^5^. RACE comprises six domains of *needle positioning*, *needle entry*, *needle driving and tissue trauma, suture placement*, *tissue approximation,* and *knot-tying* (Supplement 1)^43^. Each domain is scored on a Likert scale from one to five, and the overall score ranges from 6 to 30.

Videos showcasing the complete anastomosis tasks were shared with the raters, who assessed the task using RACE scoring. However, we adopted a detailed approach for instances where performance on specific stitches deviated from the overall task performance. In such cases, raters were instructed to provide specific RACE scores for those stitches, capturing fluctuations in performance. For example, occasionally raters observed that the proficiency in executing certain stitches by novices and residents improved midway through the task. Raters documented these improvements by noting that, from a specific time point, RACE scores elevated from one level to another. This precise method of evaluation was essential for assigning accurate RACE scores to individual stitches. This scoring approach was chosen because it is comprehensive and allows for a detailed understanding of surgical performance improvement over the course of the task. The scoring method ensures that our analysis captures both the overarching proficiency in the anastomosis procedure and the details of performance variation across different task segments.

The average of the scores given by three raters to each RACE domain was used as the actual score of that domain for each participant performing anastomosis.

### Agreement between raters’ assessments of overall performance score

Intra-class correlation coefficients (ICC) were used to assess inter-rater reliability. A two-way random effects model for absolute agreement showed a moderate degree of agreement in the raters’ overall performance scores, 0.80 with a 95% confidence interval from 0.70 to 0.84 (F92,92 = 4.35, *P* value < 0.0001). Inter-rater reliability values for the RACE domains were provided in Supplement 2.

### Overall skill levels

One of the raters (J.L.M.), who is an expert RAS surgeon, assessed the overall skill level of participants in conducting each anastomosis attempt, based on the rater’s RACE scores, into three categories: inexperienced (i.e., requires significant practice/needs improvement); competent (i.e., good/at the appropriate level), or experienced (i.e., excellent/established). These assessments were used to compare overall performance scores across overall skill levels.

### Subtasks

Five subtasks were extracted: (1) Grasping the needle; (2) positioning the needle: successfully locating the needle over tissue just before starting insertion; (3) needle driving through wrist rotation; (4) positioning the needle over the posterior plate till maximum pull out the thread; and (5) knot-tying. Each subtask’s start and end times were retrieved via accompanying recorded videos. The number of extracted samples for subtasks one to five while performing anastomosis on the plastic model were 524, 524, 534, 534, and 61; and those were 464, 464, 446, 446, and 62 for tissue anastomoses. Knot-tying subtasks were excluded from further analysis due to a small sample size.

Data for subtasks one to three were used to develop models for performance prediction in needle positioning, needle entry, needle driving and tissue trauma, respectively. Data for subtask four were used to develop models for both the suture placement and tissue approximation domains.

Eye-tracking and EEG data associated with each subtask were extracted and used to retrieve six visual and 20 EEG features, respectively.

### Machine learning regression models for RACE metrics’ scores prediction

RFR and GBR models were developed to predict RACE metrics’ scores. RFR is an ensemble learning method that combines multiple decision trees to make a final prediction. GBR is an ensemble learning algorithm that combines multiple weak learners (decision trees) to create a strong learner. The key idea of gradient boosting is to fit each new decision tree to the residual errors made by the previous tree. In this way, the new trees complement the previous ones, gradually improving the overall prediction accuracy.

We employed two approaches to partition the data for training and testing. Approach 1: 20% of the samples were chosen randomly and used as the test set. The remaining 80% of samples were used to train and validate the model using group five-fold cross-validation, repeated 10 times. The training and testing processes were repeated 10 times (bootstrapping), and the average measurements calculated from predictions for test samples were reported. Approach 2: Three participants were randomly selected, and all their samples were used as a test set, with the rest (samples from 20 remaining participants) used for training-validation. The training and testing processes were repeated 5 times. This approach used group k-fold (k = 5) cross-validation and grid search, to ensure that all samples from the same participant stay together, either in the training or test set. This method is particularly relevant in medical applications where inter-participant variability is significant and models need to generalize across different participants.

Hyperparameters of the models were tuned using grid search and cross-validation techniques. The main hyperparameters for the RFR model and the values considered for each parameter for tuning are:

- ‘n_estimators’: Number of trees in the forest. Increasing this number can improve model performance but may also lead to longer training times and a higher risk of overfitting. Range in this study: 25–300, with increments of 25.
- ‘max_depth’: Maximum depth of each decision tree. Deeper trees can capture more complex patterns but may overfit the training data. Range in this study: 1 to 41, with increments of 3.
- ‘max_features’: Maximum number of features considered for the best split. Reducing this number may help prevent overfitting but could lower model performance. Values in this study include: 5, 10, 15, 20, 26.
- ‘min_samples_leaf’: Minimum number of samples required at each leaf node. Range in this study: 1–5, with increments of 1.
- ‘min_samples_split’: Minimum number of samples required to split an internal node. Range in this study: 1–5, with increments of 2.

The main hyperparameters for GBR model and the values considered for each parameter for tuning are:

- ‘n_estimators’, ‘max_depth’ and ‘max_features’: As defined above for RFR.
- ‘learning_rate’: Influences the contribution of each tree to the final outcome. A lower rate requires more trees to achieve similar performance but can provide more robust models. Range in this study: 0.1–1, with increments of 0.1.

Early stopping is a form of regularization used to avoid overfitting when training a machine learning model, particularly with iterative methods like gradient boosting. The method involves stopping the training process if the model’s performance on a validation set does not improve for a specified number of consecutive training iterations. This method ensures that the model does not continue to learn peculiarities in the training data that do not generalize to new data. We implemented early stopping in our GBR model, stopping training if there was no improvement in validation loss within a tolerance of 0.01 over 10 consecutive iterations.

We optimized a range of key parameters through a series of trials to prevent overfitting in our RFR models. These parameters included the maximum depth of trees, the minimum number of samples required at a leaf node, the number of trees, and the number of features used for each split. Limiting the depth of each tree helps prevent the models from becoming overly complex and overfitting by learning too much detail from the training data, which may include noise. Increasing the minimum number of samples per leaf helps avoid creating overly specific leaves that represent small data subsets, thus reducing the model’s complexity. Increasing the number of trees does not lead to overfitting, but it does result in diminishing returns beyond a certain point and increases computational costs. Restricting the number of features considered for each split ensured that the trees are less likely to fit to noise in the data.

Evaluation of RFR and GBR models: Several metrics were used to evaluate the performance of regression models:

- Mean Absolute Error (MAE) measures the absolute difference between the predicted and actual values.
- R-squared (R^2^) measures the proportion of variance in the target variable that is explained by the model. R^2^ ranges from 0 to 1, with a higher value indicating better model performance.
- Root Mean Squared Error (RMSE) measures the average difference between the predicted and actual values in the same units as the target variable.

### Feature importance

The permutation importance technique, involving five repetitions, was employed to robustly estimate the importance of each feature in predicting RACE metrics in test samples using Approach 2. This method was chosen for its effectiveness in quantifying the impact of feature perturbation on model accuracy, thereby providing information about the effectiveness of features on outcomes.

The RFR and GBR models were developed in Python 3.7 using scikit-learn library.

### Statistical analyses for retrieving association between extracted features and RACE scores

Least Absolute Shrinkage and Selection Operator (LASSO) is a statistical technique that performs both variable selection and regularization to enhance the prediction accuracy and interpretability of the statistical model it produces. LASSO does this by imposing a constraint on the sum of the absolute values of the model parameters, the effect of which is to force some of the coefficient estimates to be exactly equal to zero^44^. LASSO helps prevent model overfitting because of its regularization properties. Additionally, LASSO effectively reduces multicollinearity among predictors by shrinking some coefficients to zero. The LASSO method was used for feature selection and the selected features from the LASSO model were used to develop linear mixed model (LMM) models. Additionally, the ten-fold cross-validation and grid search techniques were used to tune a key parameter of LASSO, the lambda factor, to prevent the model from overfitting. When tuning lambda, we considered a sequence of values from 1 to 50, in increments of 5, as our search space. The lambda yielding the smallest mean squared error (MSE)—determined across all 9/1 splits of training/validation datasets—was selected. A p-value less than 0.05 was considered a statistically significant predictor. The LMM models were developed in R 4.3.1.

### Distribution of overall performance scores across skill levels

An LMM was fitted for the overall performance score, where the overall skill levels were treated as three factors (levels 1: inexperienced; 2: competent; or 3: experienced), and participant ID was treated as a random effect to accommodate for repeated measurement. ANOVA was fitted to test if there was any difference in overall performance between different skill levels. A p-value less than 0.05 was considered a statistically significant difference between skill levels.

### Informed consent statement

This study was conducted in accordance with relevant guidelines and regulations approved by the Institutional Review Board (IRB; I-241913) of the Roswell Park Comprehensive Cancer Center. The IRB specifically granted a waiver for the requirement of obtaining written consent from the participants; they determined that the study posed minimal risk and that obtaining such consent was not necessary. To ensure participants are fully informed about the nature of the study, its objectives, their involvement, and their rights, all participants were provided with a detailed research study information sheet. This sheet outlined the goals of the study, the tasks participants would be involved in, and the principal investigator’s contact information. Participants were required to acknowledge receipt and understanding of this information sheet by signing it and providing verbal agreement to participate, in accordance with the IRB’s waiver of written consent.

## Results

### RACE metrics’ scores prediction models

Eye-tracking and EEG features, along with actual RACE metrics scores, were used to develop models predicting performance in anastomosis subtasks. Tables 1 and 2 show the performance of the RFR and GBR machine learning models from Approach 1 and Approach 2, respectively.

**Table 1:** Performance of RFR and GBR models developed for predicting performance scores for RACE metrics (metrics scales 1 to 5) using Approach 1.

	RFR model				GBR model			
	Needle positioning		Needle entry		Needle positioning		Needle entry	
	Plastic model	Animal tissue	Plastic model	Animal tissue	Plastic model	Animal tissue	Plastic model	Animal tissue
MAE	0.28	0.31	0.30	0.23	0.27	0.30	0.30	0.22
R^2^	0.83	0.69	0.82	0.80	0.83	0.68	0.80	0.78
RMSE	0.43	0.43	0.46	0.32	0.42	0.44	0.48	0.33

	Needle driving and tissue trauma metric							
	RFR model				GBR model			
	Plastic model		Animal tissue		Plastic model		Animal tissue	
MAE	0.35		0.28		0.32		0.28	
R^2^	0.83		0.74		0.83		0.72	
RMSE	0.50		0.42		0.49		0.43	

	RFR model				GBR model			
	Suture placement		Tissue approximation		Suture placement		Tissue approximation	
	Plastic model	Animal tissue	Plastic model	Animal tissue	Plastic model	Animal tissue	Plastic model	Animal tissue
MAE	0.22	0.35	0.37	0.37	0.23	0.35	0.37	0.36
R^2^	0.80	0.77	0.74	0.69	0.77	0.74	0.71	0.66
RMSE	0.35	0.49	0.51	0.52	0.37	0.52	0.52	0.54

The reported results represent the average outcomes from 10 repeated predictions of metric scores applied to a randomly selected 20% sample of the data (test samples).

**Table 2:** Performance of RFR and GBR models developed for predicting performance scores for RACE metrics (metrics scales 1 to 5) using Approach 2.

	RFR model				GBR model			
	Needle positioning		Needle entry		Needle positioning		Needle entry	
	Plastic model	Animal tissue	Plastic model	Animal tissue	Plastic model	Animal tissue	Plastic model	Animal tissue
MAE	0.29	0.39	0.31	0.24	0.30	0.34	0.31	0.24
R^2^	0.79	0.60	0.74	0.76	0.76	0.62	0.70	0.74
RMSE	0.47	0.52	0.54	0.35	0.50	0.48	0.57	0.37

	Needle driving and tissue trauma metric							
	RFR model				GBR model			
	Plastic model		Animal tissue		Plastic model		Animal tissue	
MAE	0.36		0.34		0.33		0.35	
R^2^	0.77		0.65		0.80		0.60	
RMSE	0.57		0.49		0.54		0.52	

	RFR model				GBR model			
	Suture placement		Tissue approximation		Suture placement		Tissue approximation	
	Plastic model	Animal tissue	Plastic model	Animal tissue	Plastic model	Animal tissue	Plastic model	Animal tissue
MAE	0.24	0.37	0.37	0.39	0.27	0.39	0.38	0.39
R^2^	0.75	0.68	0.70	0.62	0.70	0.65	0.67	0.60
RMSE	0.39	0.59	0.54	0.59	0.43	0.62	0.56	0.62

The reported results represent the average outcomes from 5 repeated predictions of metric scores applied to all samples from 3 randomly selected participants (unseen test samples).

**Figure 2:**
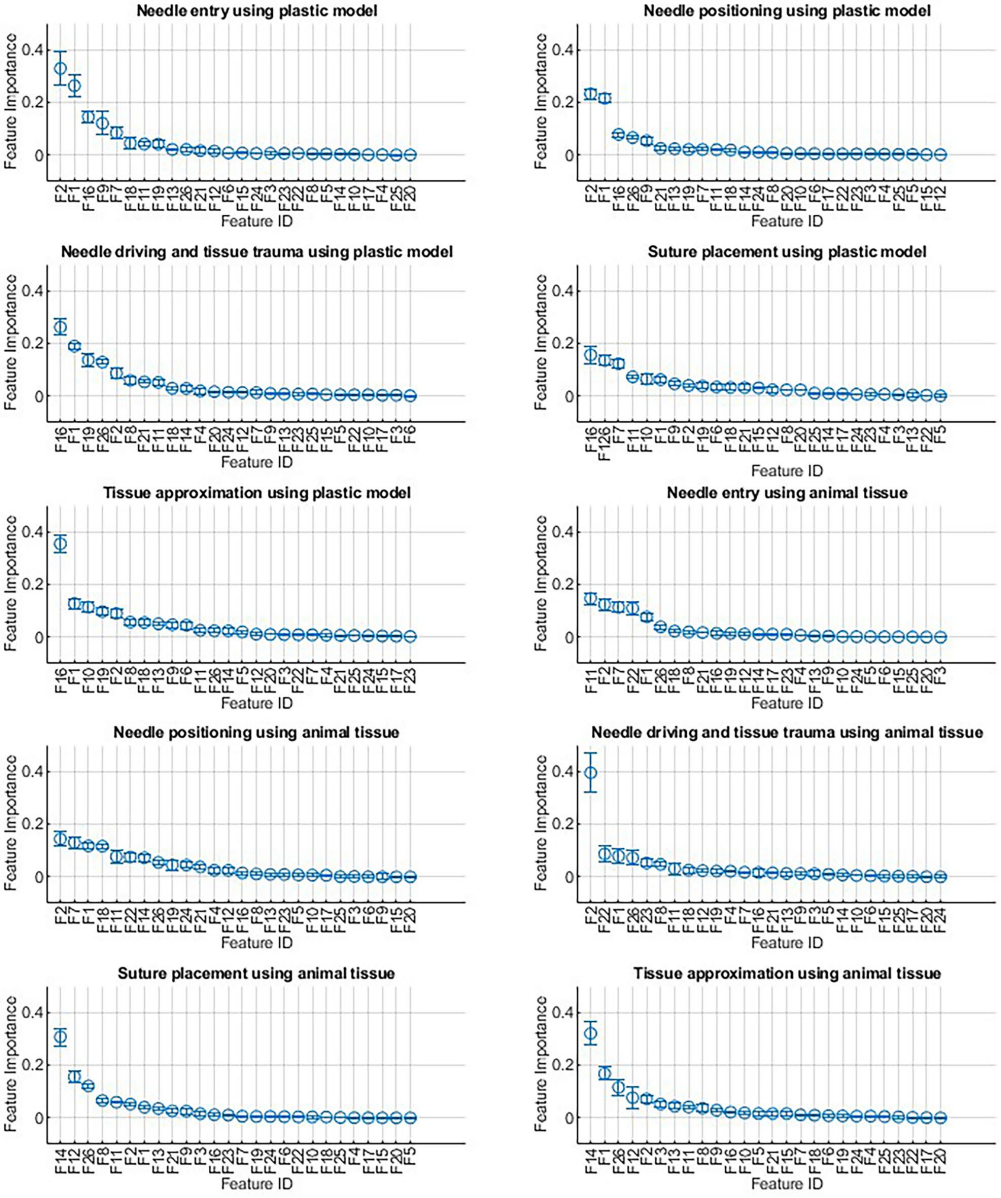
Feature importances. Representation of feature importances for RACE metrics prediction models. F1 to F26 are defined in Methods section.

In Approach 2, the importance of features for predicting RACE metrics in test samples was calculated using the permutation importance technique with five repetitions; the results for best prediction models are shown in Fig. 2.

### Relationship between EEG and eye-tracking features, and RACE scores

Samples from each respective subtask were analyzed using the LASSO feature selection method, and the selected features were used to develop an LMM model. The results derived from these LMM models are shown in Supplement 3.

### Distribution of overall performance score across overall skill levels

Average overall performance scores assessed by 3 raters were used as actual performance scores. The skill level of each anastomosis attempt was evaluated by one rater (J.L.M.) as inexperienced, competent, and experienced (Table 3). Overall performance (i.e., rater’s overall RACE score) differed significantly across the 3 skill levels (*p* value < 0.0001) and increased significantly from inexperienced to competent (*p* value = 0.0001), from competent to experienced (*p* value < 0.0001), and from inexperienced to experienced (*p* value < 0.0001).

**Table 3:** Distribution of overall performance across overall skill levels.

Skill level	Number of samples	Performance (i.e., overall RACE score)
Inexperienced	43	17.88 ± 4.04
Competent	27	22.78 ± 3.22
Experienced	22	27.55 ± 2.04

## Discussion

This study proposes the use of EEG and eye-tracking data to develop machine learning models for objective evaluation of RACE metrics for RAS anastomosis. Features extracted using EEG and eye-tracking data were used to develop models and test them rigorously. The bias effect on performance evaluation was decreased by determining actual RACE scores using the scores provided by 3 raters.

The overall performance score across different skill levels emphasizes the predictive power of the RACE metrics used. The fact that the score increased significantly from inexperienced to competent, from competent to experienced, and from inexperienced to experienced may provide evidence for a performance gradient, which supports the validity of these RACE metrics.

### The potential of EEG and eye-tracking metrics with machine learning algorithms for RACE metrics’ prediction

The results provide valuable knowledge about the feasibility of predicting RACE scores using objective metrics derived from EEG and eye-tracking data. The results support the initial hypothesis that EEG and eye-tracking patterns can serve as effective predictors of a surgeon’s performance during RAS anastomosis. The results also highlight the potential of using machine learning models, such as RFR and GBR, to make these predictions. The comparison of the machine learning models’ performances shows that both RFR and GBR have potential for predicting RACE metrics’ scores, but the choice between the two may depend on the specific subtask. The relatively moderate R^2^ values across RFR and GBR models for most RACE metrics indicate that ensemble methods like RFR and GBR are effective for this type of regression problem. Their ability to capture complex nonlinear relationships and interactions among features is critical in accurately predicting performance scores in surgical training metrics.

The RFR and GBR models show varying performance across plastic models and animal tissues, which is expected due to the differing physical properties and the way these materials react to surgical manipulation. This variation is captured well by both models, indicating they can distinguish between the two contexts effectively. The variation in model performance between plastic models and animal tissues highlights the complexity of surgical environments and the importance of tailored models for different training materials. For instance, the generally higher MAE and RMSE for animal tissues may reflect the more challenging nature of predicting performance scores in a more variable and less predictable environment.

The prediction models developed using Approach 2 for model training and testing show promise for creating objective performance evaluation tools in surgical training, especially in RAS anastomosis. The ability to predict performance scores reasonably accurate across different metrics and materials could help modify training programs to individual needs, monitor progress more effectively, and identify areas requiring improvement.

Approach 2 likely provides a better assessment of the models’ ability to generalize to unseen participants, which is crucial in clinical applications. The increased MAE and RMSE in Approach 2, especially noticeable in tasks involving animal tissues, suggest that predicting performance scores for new participants (potentially with different baseline skills or anatomical variations) is more challenging than within a mixed participant sample. The variation in R^2^ values between the two approaches, with generally lower values in Approach 2, indicates that leaving entire samples of participants out of the training set (as in Approach 2) poses a more rigorous test of model robustness against participant-specific factors.

The differences in model performance between the two approaches underscore the significance of inter-participant variability in RACE performance metrics. Approach 2, by testing on entire samples of randomly selected participants, simulates a more realistic scenario where the model is applied to entirely new individuals, which emphasizes the need for models that can accommodate or adapt to such variability. If the goal is to predict performance within a similar cohort of participants (e.g., within a single training program), Approach 1 might suffice. However, for broader applications across different populations or institutions, Approach 2’s method of testing generalization to new participants would be more indicative of real-world performance.

Figure 2 details the feature importance for prediction models. These results reveal that different predictors impact the performance of anastomosis subtasks variably, particularly in comparisons between plastic and tissue anastomosis. This complex interplay highlights the significant roles of various features, including eye-tracking and EEG-recorded brain activity in different brain regions, in predicting RACE scores. The results suggest that the brain’s ability to manage and integrate information is critical to surgical performance. Understanding the cognitive processes involved in different anastomosis subtasks would help design better surgical simulators. Simulators could be programmed to mimic the complexities of the subtasks better and provide feedback based on these objective measures.

The LMM models (Supplement 3) reveal that the diameters of the dominant and non-dominant eyes have statistically significant but opposite associations with performance on specific RACE metrics. These metrics include needle entry, suture placement, and tissue approximation using a plastic model, as well as needle positioning using animal tissue. Possible reasons for this finding include:

1) Physiological differences: There are inherent differences in the way the dominant and nondominant eyes function that are relevant to the task, especially tasks that involve depth perception or fine visual detail^45,46^ such as needle entry and needle positioning. For example, the dominant eye is often relied upon for tasks requiring fine motor skills and precision while the nondominant eye contributes to broader visual assessment and spatial awareness. The coordination of these tasks often involves both eyes, but the dominant eye plays a crucial role in guiding precise movements. This is due to its ability to provide detailed visual information, which is critical for accurate hand-eye coordination.

Exploring new training methods to balance the visual input from both eyes, particularly in professions where precise depth perception is critical (e.g., needle entry), suggests that training can reduce sensory eye dominance, potentially improving depth perception by encouraging a more balanced use of both eyes^45^.

2) Cognitive load and stress: Pupil dilation is often associated with cognitive load and stress^47^. Increased pupil size in the dominant eye may be a sign of increased stress or cognitive load, which might impair performance due to over-focus. In contrast, an increase in the nondominant eye’s pupil size might indicate an increased state of alertness that aids in performance without the negative effects of stress.

The significant role of the pupil diameter of dominant and non-dominant eyes in predicting RACE scores, as shown in Fig. 2, along with their statistically significant association with RACE scores across several anastomosis subtasks (Supplement 3), underscores the importance of incorporating ocular dominance training into RAS programs. Although current robotic surgery training modules include tasks to improve ambidexterity, there is a lack of comparable modules designed to enhance ocular dominance, particularly in improving the non-dominant eye's capability to match the dominant one.

The parietal cortex is associated with spatial awareness and processing sensory information^48,49^ and the frontal cortex is known for its role in complex decision-making and problem-solving^50,51^. The occipital cortex, primarily recognized as the visual processing center of the brain^52^, is crucial for interpreting visual data, including the identification of anatomical structures and the depth of incisions or needle insertions. Similarly, the temporal cortex is essential for the perception and interpretation of both visual and auditory information, including object recognition^53,54^, which is vital for surgeons to accurately identify different tissues, anatomical structures, and surgical tools. The importance of EEG features from these brain lobes in the performance prediction model for various anastomosis subtasks aligns with the cognitive demands of these tasks, which require advanced visual processing skills, sensory-motor integration, object recognition, and spatial awareness.

Some EEG features, such as the average integration between channels in the parietal cortex and channels in other cortices, demonstrated opposing associations with performance on different RACE metrics, such as suture placement versus needle driving and tissue trauma. The opposite directions of these coefficients—negative for suture placement and positive for needle driving and tissue trauma—suggest that the functional demands of these surgical subtasks differ. The parietal cortex, involved in sensory processing and integrating sensory information with motor commands^48,49^, may play a more beneficial role in the coordination required for needle driving and managing tissue trauma than in suture placement. This finding could imply that different cognitive or neural mechanisms are emphasized in different tasks. For instance, suture placement might require more precise, localized control that is hindered by excessive integration, which could lead to the overgeneralization of motor commands or sensory inputs. In contrast, needle driving and tissue handling might benefit from broader integrative processing to handle complex spatial and textural information.

### Strengths of this study and practical implications of the findings

Studies assessing performance using eye-tracking and EEG are available, and their findings are promising^20,21,55,56^. However, the majority of those studies have weaknesses that include: (1) studying more basic tasks with levels of complexity that are not comparable to RAS anastomosis using plastic model and animal tissue; (2) using data from novices rather than participants with a variety of RAS experience; (3) evaluating actual performance scores provided by a single rater; (4) or using a single performance score for all sections of a procedure^57^.

To objectively assess the performance of anastomosis subtasks, current study proposes models using EEG and eye-tracking features and actual RACE scores assessed by 3 raters. The developed models might be transferable to clinical settings because: (1) 3 raters assessed performance using RACE metrics; (2) models were trained with data from 23 participants with varying levels of RAS experience; and (3) the anastomosis models were closer to the actual operative procedure.

The bias in performance evaluation was reduced by averaging RACE scores from three raters. The application of such relatively unbiased performance evaluation techniques will improve RAS training and skill acquisition by providing quantitative feedback to trainees regarding their performance in conducting anastomosis subtasks. Poor performance in conducting anastomosis results in patient complications that impair quality of life and results in additional procedures that are costly for the patients, insurers, and hospitals. RAS trainees’ skills can be improved before performing this procedure on a patient by giving them feedback on how well they performed the subtasks of anastomosis.

The proposed models have the potential to provide trainees with feedback on their performance in various RAS anastomosis subtasks. EEG and eye-tracking data are signals and have the potential to be collected and analyzed in real-time with less computing cost compared to other types of data such as surgical videos^58^. Personalized learning and eventually automated performance feedback may be made possible by developing machine learning models that are trained using EEG and eye-tracking features^59^.

### Clinical Implications and educational value of the findings

This study provides a method for objectively assessing performance in critical anastomosis subtasks, such as needle positioning and suture placement. By identifying specific areas where a surgeon may require additional training, findings offer a targeted approach that could streamline the training process, reduce learning costs, and potentially increase the annual number of proficiently trained surgeons. Over time, this could lead to a reduction in procedural errors, enhanced patient outcomes, and improved overall efficiency of surgical operations. The effectiveness of our models across different materials (plastic model and animal tissue) demonstrates their versatility and broad applicability in various surgical training scenarios.

Findings of this study highlights the potential benefits of integrating machine learning and physiological data (eye-tracking and EEG) into the surgical trainee evaluation process. By quantitatively measuring performance in anastomosis subtasks, developed models provide educators with a novel perspective for assessing and enhancing trainees’ competencies. This method improves the objectivity of performance assessments and supports personalized feedback, addressing individual areas for improvement. Additionally, our findings suggest the necessity of incorporating tasks to improve ocular dominance into existing RAS training programs.

### Limitations of this study and future plan

Although this study’s results are encouraging, there are several limitations. More participants from various institutes and specialties are required to generalize the validity of the developed models. The start and end time of anastomosis subtasks were extracted manually from operative videos. A more efficient method for subtask extraction is needed. Additionally, future research should explore how participants’ backgrounds influence eye metrics and EEG data during RAS procedures. Such analysis would enhance our understanding of the diverse factors that affect surgical skill acquisition and inform more tailored training approaches. Integrating other machine learning techniques, such as deep learning, could offer understandings about further improving model accuracy and generalizability. Incorporating live surgical videos and employing laboratory-based EEG and eye-tracking metrics to predict live RACE scores could enhance this study by advancing our understanding of surgical competence.

## Conclusion

This study presents compelling evidence for the utility of EEG and eye-tracking features for evaluating RACE metrics in anastomosis subtasks. This study also demonstrates the potential of machine learning models to use these features for reasonably accurate performance prediction. Although promising, these findings require replication and validation across various surgical procedures before these predictive models can be adopted for surgical training.

### Supplementary Information

Supplementary Information 1.

Supplementary Information 2.

Supplementary Information 3.

## Data Availability

The datasets generated during and/or analyzed during the current study are available from the corresponding author (SBS) upon reasonable request.
