# Supplementary material for: Prediction of Robotic Anastomosis Competency Evaluation (RACE) metrics during vesico-urethral anastomosis using electroencephalography, eye-tracking, and machine learning
Source: Sci Rep. 2024 Jun 25;14:14611. doi: 10.1038/s41598-024-65648-3 (PMC11199555; doi:10.1038/s41598-024-65648-3)
Supplement: Supplementary file 1 — Supplementary Information 1. [file 41598_2024_65648_MOESM1_ESM.docx]

**Supplement 1.** Domains of the Robotic Anastomosis Competency Evaluation metrics (RACE) tool [43].


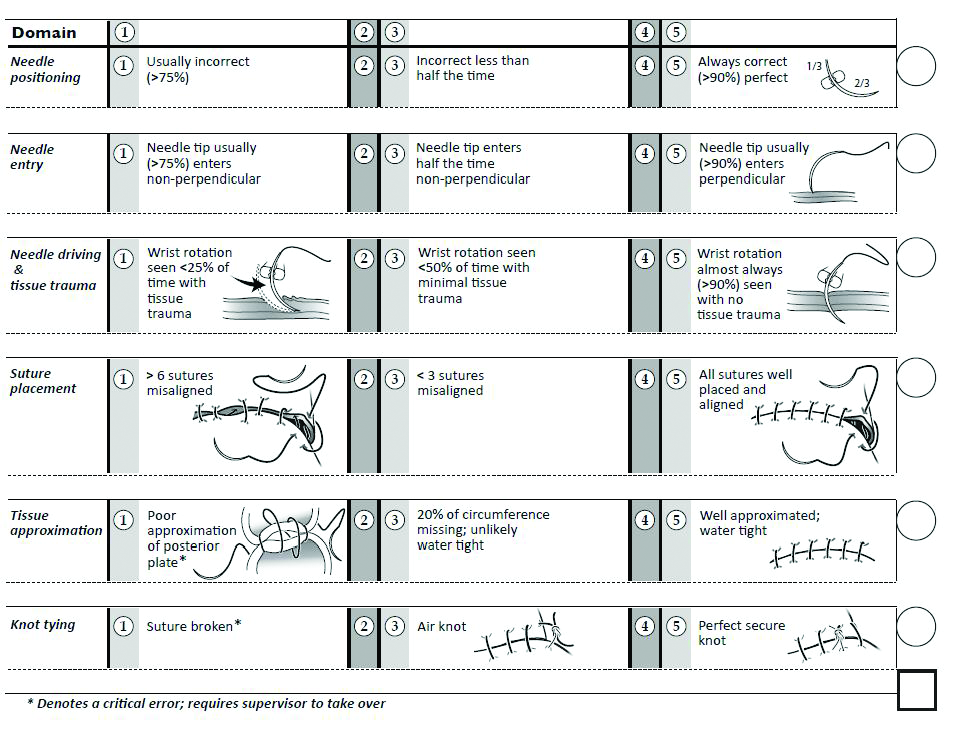


Figure 1 Robotic Anastomosis Competency Evaluation form.
