# Supplementary material for: Prediction of Robotic Anastomosis Competency Evaluation (RACE) metrics during vesico-urethral anastomosis using electroencephalography, eye-tracking, and machine learning
Source: Sci Rep. 2024 Jun 25;14:14611. doi: 10.1038/s41598-024-65648-3 (PMC11199555; doi:10.1038/s41598-024-65648-3)
Supplement: Supplementary file 2 — Supplementary Information 2. [file 41598_2024_65648_MOESM2_ESM.docx]

**Supplement 2:** The Intra-class correlation coefficients (ICC) values for each Robotic Anastomosis Competency Evaluation (RACE) domain

| **RACE metric** | **ICC** | **Lower bound** | **Upper bound** |
| --- | --- | --- | --- |
| Needle positioning performed using plastic model | 0.74 | 0.53 | 0.86 |
| Needle positioning performed using animal tissue | 0.60 | 0. 54 | 0.81 |
| Needle entry performed using plastic model | 0.70 | 0.57 | 0.81 |
| Needle entry performed using animal tissue | 0.65 | 0.50 | 0.76 |
| Needle driving and tissue trauma performed using plastic model | 0.80 | 0.62 | 0.88 |
| Needle driving and tissue trauma performed using animal tissue | 0.71 | 0.52 | 0.84 |
| Suture placement performed using plastic model | 0.61 | 0.56 | 0.77 |
| Suture placement performed using animal tissue | 0.76 | 0.65 | 0.87 |
| Tissue approximation performed using plastic model | 0.70 | 0.57 | 0.81 |
| Tissue approximation performed using animal tissue | 0.72 | 0.53 | 0.85 |
