# Supplementary material for: Prediction of Robotic Anastomosis Competency Evaluation (RACE) metrics during vesico-urethral anastomosis using electroencephalography, eye-tracking, and machine learning
Source: Sci Rep. 2024 Jun 25;14:14611. doi: 10.1038/s41598-024-65648-3 (PMC11199555; doi:10.1038/s41598-024-65648-3)
Supplement: Supplementary file 3 — Supplementary Information 3. [file 41598_2024_65648_MOESM3_ESM.docx]

**Supplement 3:** Results of linear mixed-effect model (LMM), using Least Absolute Shrinkage and Selection Operator (LASSO) feature selection technique, for performance evaluation

Table 1 Results of LMM, using lasso feature selection technique, for performance evaluation at the needle entry using plastic models

| **Needle entry, plastic anastomosis** | | |
| --- | --- | --- |
| *Predictors* | *Coefficients* | *p-values* |
| Average pupil diameter, nondominant eye | 0.151 | **0.027** |
| Average pupil diameter, dominant eye | -0.166 | **0.008** |
| Entropy of pupil diameter, nondominant eye | 0.065 | 0.109 |
| Entropy of pupil diameter, dominant eye | 0.032 | 0.377 |
| rate of fixation time points | 0.023 | 0.586 |
| rate of saccade time points | 0.029 | 0.516 |
| Average temporal network flexibility of channels in parietal cortex | -0.028 | 0.349 |
| Average integration between channels in parietal cortex and channels in other cortices | 0.119 | **0.030** |
| Average recruitment of channels in parietal cortex | 0.030 | 0.370 |
| Average search information for channels in parietal cortex | -0.394 | **<0.001** |
| Average temporal network flexibility of channels in frontal cortex | -0.003 | 0.894 |
| Average integration between channels in frontal cortex and channels in other cortices | 0.187 | **<0.001** |
| Average recruitment of channels in frontal cortex | -0.098 | **<0.001** |
| Average search information for channels in frontal cortex | 0.175 | 0.073 |
| Average strength of channels in frontal cortex | -0.399 | **<0.001** |
| Average temporal network flexibility of channels in occipital cortex | 0.011 | 0.607 |
| Average integration between channels in occipital cortex and channels from other cortices | -0.157 | **<0.001** |
| Average recruitment of channels in occipital cortex | -0.008 | 0.683 |
| Average search information for channels in occipital cortex | -0.194 | **0.028** |
| Average strength of channels in occipital cortex | -0.055 | 0.451 |
| Average temporal network flexibility of channels in temporal cortex | 0.023 | 0.386 |
| Average integration between channels in temporal cortex and channels from other cortices | -0.067 | 0.248 |
| Average recruitment of channels in temporal cortex | 0.009 | 0.651 |
| Average search information for channels in temporal cortex | 0.206 | 0.076 |
| Average strength of channels in temporal cortex | 0.457 | **<0.001** |
| Number of samples: 524 |  | |
| R^2^: 0.80; MAE: 0.39; RMSE: 0.48 |  | |

Table 2 Results of LMM, using lasso feature selection technique, for performance evaluation at the needle positioning using plastic models

| **Needle positioning, plastic anastomosis** | | |
| --- | --- | --- |
| *Predictors* | *Coefficients* | *p-values* |
| Average pupil diameter, nondominant eye | 0.096 | 0.139 |
| Average pupil diameter, dominant eye | -0.193 | **0.001** |
| Entropy of pupil diameter, nondominant eye | 0.025 | 0.513 |
| Entropy of pupil diameter, dominant eye | 0.048 | 0.164 |
| rate of fixation time points | 0.041 | 0.319 |
| rate of saccade time points | 0.031 | 0.456 |
| Average temporal network flexibility of channels in parietal cortex | -0.031 | 0.273 |
| Average integration between channels in parietal cortex and channels in other cortices | 0.029 | 0.576 |
| Average recruitment of channels in parietal cortex | -0.037 | 0.241 |
| Average search information for channels in parietal cortex | -0.139 | 0.117 |
| Average temporal network flexibility of channels in frontal cortex | -0.029 | 0.240 |
| Average integration between channels in frontal cortex and channels in other cortices | 0.159 | **<0.001** |
| Average recruitment of channels in frontal cortex | -0.094 | **<0.001** |
| Average search information for channels in frontal cortex | 0.041 | 0.657 |
| Average strength of channels in frontal cortex | -0.456 | **<0.001** |
| Average temporal network flexibility of channels in occipital cortex | 0.006 | 0.795 |
| Average integration between channels in occipital cortex and channels from other cortices | -0.083 | 0.050 |
| Average recruitment of channels in occipital cortex | -0.041 | **0.034** |
| Average search information for channels in occipital cortex | -0.129 | 0.124 |
| Average strength of channels in occipital cortex | -0.027 | 0.695 |
| Average temporal network flexibility of channels in temporal cortex | 0.039 | 0.121 |
| Average integration between channels in temporal cortex and channels from other cortices | 0.006 | 0.917 |
| Average recruitment of channels in temporal cortex | 0.058 | **0.001** |
| Average search information for channels in temporal cortex | 0.056 | 0.612 |
| Average strength of channels in temporal cortex | 0.424 | **<0.001** |
| Number of samples: 524 |  | |
| R^2^: 0.87; MAE: 0.30; RMSE: 0.38 |  | |

Table 3 Results of LMM, using lasso feature selection technique, for performance evaluation at the needle driving and tissue trauma using plastic models

| **Needle driving and tissue trauma, plastic anastomosis** | | |
| --- | --- | --- |
| *Predictors* | *Coefficients* | *p-values* |
| Average pupil diameter, nondominant eye | 0.133 | 0.069 |
| Average pupil diameter, dominant eye | -0.150 | **0.044** |
| Entropy of pupil diameter, nondominant eye | 0.207 | **<0.001** |
| Entropy of pupil diameter, dominant eye | -0.142 | **<0.001** |
| rate of fixation time points | -0.058 | 0.397 |
| rate of saccade time points | -0.106 | 0.131 |
| Average integration between channels in parietal cortex and channels in other cortices | 0.208 | **<0.001** |
| Average search information for channels in parietal cortex | -0.525 | **<0.001** |
| Average integration between channels in frontal cortex and channels in other cortices | -0.002 | 0.965 |
| Average search information for channels in frontal cortex | -0.008 | 0.943 |
| Average strength of channels in frontal cortex | -0.294 | 0.005 |
| Average temporal network flexibility of channels in occipital cortex | 0.017 | 0.584 |
| Average integration between channels in occipital cortex and channels from other cortices | -0.344 | **<0.001** |
| Average search information for channels in occipital cortex | 0.110 | 0.238 |
| Average strength of channels in occipital cortex | -0.341 | **<0.001** |
| Average temporal network flexibility of channels in temporal cortex | -0.008 | 0.802 |
| Average integration between channels in temporal cortex and channels from other cortices | 0.013 | 0.871 |
| Average search information for channels in temporal cortex | 0.324 | **0.014** |
| Average strength of channels in temporal cortex | 0.526 | **<0.001** |
| Number of samples: 534 |  | |
| R^2^: 0.69; MAE: 0.53; RMSE: 0.66 |  | |

Table 4 Results of LMM, using lasso feature selection technique, for performance evaluation at the suture placement using plastic models

| **Suture placement, plastic anastomosis** | | |
| --- | --- | --- |
| *Predictors* | *Coefficients* | *p-values* |
| Average pupil diameter, nondominant eye | 0.359 | **<0.001** |
| Average pupil diameter, dominant eye | -0.310 | **<0.001** |
| Entropy of pupil diameter, nondominant eye | -0.011 | 0.764 |
| Entropy of pupil diameter, dominant eye | 0.081 | **0.008** |
| rate of fixation time points | 0.234 | **<0.001** |
| rate of saccade time points | 0.187 | **0.001** |
| Average temporal network flexibility of channels in parietal cortex | -0.078 | **0.034** |
| Average integration between channels in parietal cortex and channels in other cortices | -0.222 | **<0.001** |
| Average recruitment of channels in parietal cortex | -0.157 | **<0.001** |
| Average search information for channels in parietal cortex | 0.103 | 0.294 |
| Average strength of channels in parietal cortex | -0.278 | **<0.001** |
| Average temporal network flexibility of channels in frontal cortex | 0.015 | 0.630 |
| Average integration between channels in frontal cortex and channels in other cortices | 0.048 | 0.330 |
| Average recruitment of channels in frontal cortex | 0.001 | 0.971 |
| Average search information for channels in frontal cortex | -0.609 | **<0.001** |
| Average temporal network flexibility of channels in occipital cortex | 0.006 | 0.831 |
| Average integration between channels in occipital cortex and channels from other cortices | -0.097 | **0.044** |
| Average recruitment of channels in occipital cortex | 0.086 | **0.001** |
| Average search information for channels in occipital cortex | 0.042 | 0.592 |
| Average strength of channels in occipital cortex | -0.107 | 0.129 |
| Average temporal network flexibility of channels in temporal cortex | 0.006 | 0.851 |
| Average integration between channels in temporal cortex and channels from other cortices | 0.233 | **<0.001** |
| Average recruitment of channels in temporal cortex | -0.039 | 0.054 |
| Average search information for channels in temporal cortex | 0.359 | **0.001** |
| Average strength of channels in temporal cortex | 0.148 | 0.084 |
| Number of samples: 534 |  | |
| R^2^: 0.70; MAE: 0.32 RMSE: 0.43 |  | |

Table 5 Results of LMM, using lasso feature selection technique, for performance evaluation at the tissue approximation using plastic models

| **Tissue approximation, plastic anastomosis** | | |
| --- | --- | --- |
| *Predictors* | *Coefficients* | *p-values* |
| Average pupil diameter, nondominant eye | 0.641 | **<0.001** |
| Average pupil diameter, dominant eye | -0.482 | **<0.001** |
| Entropy of pupil diameter, nondominant eye | 0.135 | **0.010** |
| Entropy of pupil diameter, dominant eye | -0.067 | 0.113 |
| rate of fixation time points | 0.231 | **0.004** |
| rate of saccade time points | 0.134 | 0.102 |
| Average temporal network flexibility of channels in parietal cortex | -0.092 | 0.071 |
| Average integration between channels in parietal cortex and channels in other cortices | -0.186 | **0.035** |
| Average recruitment of channels in parietal cortex | -0.009 | 0.864 |
| Average search information for channels in parietal cortex | -0.096 | 0.495 |
| Average strength of channels in parietal cortex | 0.015 | 0.903 |
| Average temporal network flexibility of channels in frontal cortex | 0.124 | **0.004** |
| Average integration between channels in frontal cortex and channels in other cortices | 0.161 | **0.022** |
| Average recruitment of channels in frontal cortex | -0.060 | 0.053 |
| Average search information for channels in frontal cortex | -0.533 | **<0.001** |
| Average strength of channels in frontal cortex | 0.113 | 0.402 |
| Average temporal network flexibility of channels in occipital cortex | 0.077 | **0.041** |
| Average integration between channels in occipital cortex and channels from other cortices | -0.176 | **0.009** |
| Average recruitment of channels in occipital cortex | 0.196 | **<0.001** |
| Average search information for channels in occipital cortex | 0.096 | 0.380 |
| Average strength of channels in occipital cortex | -0.338 | **0.001** |
| Average temporal network flexibility of channels in temporal cortex | -0.024 | 0.589 |
| Average integration between channels in temporal cortex and channels from other cortices | 0.065 | 0.496 |
| Average search information for channels in temporal cortex | 0.511 | **0.001** |
| Average strength of channels in temporal cortex | 0.213 | 0.105 |
| Number of samples: 534 |  | |
| R^2^: 0.40; MAE: 0.62 RMSE: 0.78 |  | |

Table 6 Results of LMM, using lasso feature selection technique, for performance evaluation at the needle entry using animal tissue

| **Needle entry, tissue anastomosis** | | |
| --- | --- | --- |
| *Predictors* | *Coefficients* | *p-values* |
| Average pupil diameter, nondominant eye | 0.075 | 0.334 |
| Average pupil diameter, dominant eye | 0.085 | 0.290 |
| Entropy of pupil diameter, nondominant eye | 0.065 | **0.024** |
| Entropy of pupil diameter, dominant eye | -0.046 | 0.089 |
| rate of fixation time points | 0.032 | 0.454 |
| rate of saccade time points | 0.048 | 0.244 |
| Average temporal network flexibility of channels in parietal cortex | 0.011 | 0.615 |
| Average integration between channels in parietal cortex and channels in other cortices | -0.049 | 0.213 |
| Average recruitment of channels in parietal cortex | 0.029 | 0.093 |
| Average search information for channels in parietal cortex | 0.008 | 0.892 |
| Average strength of channels in parietal cortex | 0.090 | **0.035** |
| Average temporal network flexibility of channels in frontal cortex | 0.037 | **0.031** |
| Average integration between channels in frontal cortex and channels in other cortices | -0.049 | 0.142 |
| Average recruitment of channels in frontal cortex | 0.031 | 0.062 |
| Average search information for channels in frontal cortex | -0.065 | 0.300 |
| Average strength of channels in frontal cortex | -0.034 | 0.468 |
| Average temporal network flexibility of channels in occipital cortex | 0.026 | 0.159 |
| Average recruitment of channels in occipital cortex | -0.060 | **0.003** |
| Average search information for channels in occipital cortex | 0.050 | 0.342 |
| Average strength of channels in occipital cortex | 0.055 | 0.221 |
| Average temporal network flexibility of channels in temporal cortex | -0.034 | 0.161 |
| Average integration between channels in temporal cortex and channels from other cortices | 0.075 | 0.086 |
| Average recruitment of channels in temporal cortex | -0.008 | 0.608 |
| Average search information for channels in temporal cortex | 0.013 | 0.869 |
| Average strength of channels in temporal cortex | -0.032 | 0.638 |
| Number of samples: 464 |  | |
| R^2^: 0.62; MAE: 0.34; RMSE: 0.43 |  | |

Table 7 Results of LMM, using lasso feature selection technique, for performance evaluation at the needle positioning using animal tissue

| **Needle positioning, tissue anastomosis** | | |
| --- | --- | --- |
| *Predictors* | *Coefficients* | *p-values* |
| Average pupil diameter, nondominant eye | -0.324 | **0.009** |
| Average pupil diameter, dominant eye | 0.761 | **<0.001** |
| Entropy of pupil diameter, nondominant eye | 0.006 | 0.892 |
| Entropy of pupil diameter, dominant eye | -0.041 | 0.343 |
| rate of fixation time points | 0.089 | 0.184 |
| rate of saccade time points | 0.078 | 0.239 |
| Average temporal network flexibility of channels in parietal cortex | 0.048 | 0.179 |
| Average integration between channels in parietal cortex and channels in other cortices | 0.203 | **0.005** |
| Average recruitment of channels in parietal cortex | -0.024 | 0.380 |
| Average search information for channels in parietal cortex | 0.001 | 0.995 |
| Average strength of channels in parietal cortex | -0.035 | 0.606 |
| Average temporal network flexibility of channels in frontal cortex | 0.011 | 0.682 |
| Average integration between channels in frontal cortex and channels in other cortices | -0.025 | 0.647 |
| Average recruitment of channels in frontal cortex | 0.014 | 0.586 |
| Average search information for channels in frontal cortex | 0.080 | 0.417 |
| Average strength of channels in frontal cortex | 0.023 | 0.757 |
| Average temporal network flexibility of channels in occipital cortex | -0.015 | 0.624 |
| Average integration between channels in occipital cortex and channels from other cortices | -0.184 | **0.001** |
| Average recruitment of channels in occipital cortex | -0.031 | 0.342 |
| Average strength of channels in occipital cortex | -0.020 | 0.756 |
| Average temporal network flexibility of channels in temporal cortex | -0.011 | 0.770 |
| Average integration between channels in temporal cortex and channels from other cortices | 0.053 | 0.454 |
| Average recruitment of channels in temporal cortex | -0.068 | **0.004** |
| Average search information for channels in temporal cortex | -0.028 | 0.808 |
| Average strength of channels in temporal cortex | 0.034 | 0.739 |
| Number of samples: 464 |  | |
| R^2^: 0.52; MAE: 0.45; RMSE: 0.54 |  | |

Table 8 Results of LMM, using lasso feature selection technique, for performance evaluation at the needle driving and tissue trauma using animal tissue

| **Needle driving and tissue trauma, tissue anastomosis** | | |
| --- | --- | --- |
| *Predictors* | *Coefficients* | *p-values* |
| Average pupil diameter, nondominant eye | -0.212 | 0.118 |
| Average pupil diameter, dominant eye | 0.486 | **0.001** |
| Entropy of pupil diameter, nondominant eye | -0.014 | 0.714 |
| Entropy of pupil diameter, dominant eye | -0.101 | **0.005** |
| rate of fixation time points | 0.025 | 0.738 |
| rate of saccade time points | 0.039 | 0.596 |
| Average temporal network flexibility of channels in parietal cortex | -0.063 | 0.154 |
| Average integration between channels in parietal cortex and channels in other cortices | 0.144 | 0.073 |
| Average recruitment of channels in parietal cortex | -0.054 | 0.077 |
| Average search information for channels in parietal cortex | -0.196 | 0.063 |
| Average strength of channels in parietal cortex | 0.024 | 0.744 |
| Average temporal network flexibility of channels in frontal cortex | -0.004 | 0.916 |
| Average integration between channels in frontal cortex and channels in other cortices | -0.072 | 0.238 |
| Average recruitment of channels in frontal cortex | 0.020 | 0.491 |
| Average search information for channels in frontal cortex | -0.011 | 0.907 |
| Average strength of channels in frontal cortex | -0.043 | 0.551 |
| Average temporal network flexibility of channels in occipital cortex | 0.051 | 0.117 |
| Average integration between channels in occipital cortex and channels from other cortices | -0.175 | **0.003** |
| Average search information for channels in occipital cortex | 0.005 | 0.944 |
| Average strength of channels in occipital cortex | 0.011 | 0.878 |
| Average temporal network flexibility of channels in temporal cortex | 0.034 | 0.439 |
| Average integration between channels in temporal cortex and channels from other cortices | 0.162 | **0.041** |
| Average recruitment of channels in temporal cortex | -0.058 | **0.033** |
| Average search information for channels in temporal cortex | 0.254 | **0.030** |
| Average strength of channels in temporal cortex | 0.012 | 0.909 |
| Number of samples: 446 |  | |
| R^2^: 0.43; MAE: 0.52; RMSE: 0.62 |  | |

Table 9 Results of LMM, using lasso feature selection technique, for performance evaluation at the suture placement using animal tissue

| **Suture placement, tissue anastomosis** | | |
| --- | --- | --- |
| *Predictors* | *Coefficients* | *p-values* |
| Average pupil diameter, nondominant eye | 0.122 | 0.349 |
| Average pupil diameter, dominant eye | -0.005 | 0.974 |
| Entropy of pupil diameter, nondominant eye | 0.054 | 0.137 |
| Entropy of pupil diameter, dominant eye | -0.168 | **<0.001** |
| rate of fixation time points | -0.105 | 0.151 |
| rate of saccade time points | -0.039 | 0.585 |
| Average temporal network flexibility of channels in parietal cortex | -0.030 | 0.482 |
| Average integration between channels in parietal cortex and channels in other cortices | 0.126 | **0.030** |
| Average recruitment of channels in parietal cortex | -0.077 | **0.007** |
| Average search information for channels in parietal cortex | -0.042 | 0.670 |
| Average strength of channels in parietal cortex | 0.100 | 0.159 |
| Average temporal network flexibility of channels in frontal cortex | 0.083 | **0.013** |
| Average recruitment of channels in frontal cortex | 0.022 | 0.421 |
| Average strength of channels in frontal cortex | -0.031 | 0.648 |
| Average temporal network flexibility of channels in occipital cortex | -0.019 | 0.527 |
| Average integration between channels in occipital cortex and channels from other cortices | -0.037 | 0.506 |
| Average recruitment of channels in occipital cortex | -0.061 | 0.078 |
| Average search information for channels in occipital cortex | -0.080 | 0.284 |
| Average strength of channels in occipital cortex | -0.020 | 0.772 |
| Average temporal network flexibility of channels in temporal cortex | 0.032 | 0.427 |
| Average recruitment of channels in temporal cortex | -0.048 | 0.073 |
| Average search information for channels in temporal cortex | 0.209 | **0.033** |
| Average strength of channels in temporal cortex | -0.024 | 0.810 |
| Number of samples: 446 |  | |
| R^2^: 0.68 MAE: 0.49; RMSE: 0.59 |  | |

Table 10 Results of LMM, using lasso feature selection technique, for performance evaluation at the tissue approximation using animal tissue

| **Tissue approximation, tissue anastomosis** | | |
| --- | --- | --- |
| *Predictors* | *Coefficients* | *p-values* |
| Average pupil diameter, nondominant eye | 0.054 | 0.684 |
| Average pupil diameter, dominant eye | 0.251 | 0.077 |
| Entropy of pupil diameter, nondominant eye | 0.020 | 0.580 |
| Entropy of pupil diameter, dominant eye | -0.070 | **0.041** |
| rate of fixation time points | -0.177 | **0.014** |
| rate of saccade time points | -0.149 | **0.037** |
| Average temporal network flexibility of channels in parietal cortex | -0.044 | 0.299 |
| Average integration between channels in parietal cortex and channels in other cortices | 0.093 | 0.227 |
| Average recruitment of channels in parietal cortex | -0.141 | **<0.001** |
| Average search information for channels in parietal cortex | -0.237 | **0.020** |
| Average strength of channels in parietal cortex | -0.028 | 0.691 |
| Average temporal network flexibility of channels in frontal cortex | 0.095 | **0.004** |
| Average integration between channels in frontal cortex and channels in other cortices | 0.107 | 0.084 |
| Average recruitment of channels in frontal cortex | -0.009 | 0.759 |
| Average search information for channels in frontal cortex | 0.033 | 0.716 |
| Average strength of channels in frontal cortex | 0.024 | 0.733 |
| Average temporal network flexibility of channels in occipital cortex | -0.006 | 0.840 |
| Average integration between channels in occipital cortex and channels from other cortices | -0.133 | **0.022** |
| Average recruitment of channels in occipital cortex | 0.044 | 0.224 |
|  | -0.034 | 0.597 |
| Average strength of channels in occipital cortex | 0.019 | 0.657 |
| Average temporal network flexibility of channels in temporal cortex | 0.016 | 0.834 |
| Average integration between channels in temporal cortex and channels from other cortices | 0.000 | 0.992 |
| Average recruitment of channels in temporal cortex (24) | 0.228 | **0.030** |
| Average search information for channels in temporal cortex | 0.009 | 0.927 |
| Average strength of channels in temporal cortex | 0.054 | 0.684 |
| Number of samples: 446 |  | |
| R^2^: 0.84; MAE: 0.28; RMSE: 0.38 |  | |
